# Protective Role of Dietary Curcumin in the Prevention of the Oxidative Stress Induced by Chronic Alcohol with respect to Hepatic Injury and Antiatherogenic Markers

**DOI:** 10.1155/2016/5017460

**Published:** 2016-01-05

**Authors:** Ravi Varatharajalu, Mamatha Garige, Leslie C. Leckey, Karina Reyes-Gordillo, Ruchi Shah, M. Raj Lakshman

**Affiliations:** Lipid Research Laboratory, VA Medical Center and Department of Biochemistry and Molecular Medicine, The George Washington University, Washington, DC 20422, USA

## Abstract

Curcumin, an antioxidant compound found in Asian spices, was evaluated for its protective effects against ethanol-induced hepatosteatosis, liver injury, antiatherogenic markers, and antioxidant status in rats fed with Lieber-deCarli low menhaden (2.7% of total calories from *ω*-3 polyunsaturated fatty acids (PUFA)) and Lieber-deCarli high menhaden (13.8% of total calories from *ω*-3 PUFA) alcohol-liquid (5%) diets supplemented with or without curcumin (150 mg/kg/day) for 8 weeks. Treatment with curcumin protected against high *ω*-3 PUFA and ethanol-induced hepatosteatosis and increase in liver injury markers, alanine aminotransferase, and aspartate aminotransferase. Curcumin upregulated paraoxonase 1 (PON1) mRNA and caused significant increase in serum PON1 and homocysteine thiolactonase activities as compared to high *ω*-3 PUFA and ethanol group. Moreover, treatment with curcumin protected against ethanol-induced oxidative stress by increasing the antioxidant glutathione and decreasing the lipid peroxidation adduct 4-hydroxynonenal. These results strongly suggest that chronic ethanol in combination with high *ω*-3 PUFA exacerbated hepatosteatosis and liver injury and adversely decreases antiatherogenic markers due to increased oxidative stress and depletion of glutathione. Curcumin supplementation significantly prevented these deleterious actions of chronic ethanol and high *ω*-3 PUFA. Therefore, we conclude that curcumin may have therapeutic potential to protect against chronic alcohol-induced liver injury and atherosclerosis.

## 1. Introduction

Chronic alcohol consumption leads to alcoholic hepatosteatosis and consequently to inflammation, necrosis, fibrosis, and finally cirrhosis that afflicts millions worldwide and is one of the major causes of mortality in developed countries [1]. Liver alcohol dehydrogenase is primarily responsible for the oxidation of ethanol to acetaldehyde as well as hydroxyl radicals leading to the generation of reactive oxygen species (ROS). In addition, chronic ethanol-induced cytochrome P4502E1 (CYP2E1) mediated oxidation of ethanol also produces a state of oxidative stress by generating ROS within the cells including the *α*,*β*-unsaturated aldehyde, 4-hydroxy-2-nonenal (4-HNE) that may be more harmful than ROS because it has a longer half-life and can easily diffuse into cellular membranes [2]. The 4-HNE, in turn, is likely to play a major role for the progression of alcoholic fatty liver and liver disease. Whereas high *ω*-3 PUFA fish oil diet (~14% of dietary calories from *ω*-3 PUFA) causes severe hepatosteatosis liver injury [3], we showed that *ω*-3 PUFA fish oil diet (~2.7% of dietary calories from *ω*-3 PUFA) had protective effects [4].

Excess of ROS in alcoholics causes increased low density lipoproteins (LDL) oxidation that is avidly scavenged by macrophages leading to their transformation to foam cells that are atherogenic. Further, oxidized LDL is capable of stimulating endothelial cells to express adhesion molecules, which attract excess of foamy macrophages under the subendothelial layer, thereby promoting the seeding process of plaque formation. Incidence of atherosclerosis is high in chronic alcoholics due to higher level of ROS [5], homocysteine, and homocysteine thiolactone (HTL) [6]. Antiatherogenic enzyme, paraoxonase 1 (PON1), is predominantly synthesized in the liver and secreted into circulation, where it avidly binds and becomes an integral part of high density lipoproteins (HDL) [7]. Antiatherosclerotic role of PON1 is to inhibit the oxidation of LDL as well as to inactivate oxidized lipid molecules by hydrolyzing cholesteryl linoleate hydroxide (CL-OH) and cholesteryl linoleate hydroperoxide (CL-OOH) groups by its esterase activity, thus facilitating reverse cholesterol transport (RCT) from macrophages to the liver for degradation [8, 9]. Other crucial function of PON1 is to hydrolyze highly toxic homocysteine thiolactone ultimately to methionine by methionine synthase pathway [10, 11].

A number of etiological agents of liver disease such as smoking, alcohol, high fat and high cholesterol, high polyunsaturated fatty acids (PUFA), lipopolysaccharide (LPS), iron, homocysteine, and HTL, ROS, and PON1 gene polymorphism adversely affect PON1 activity leading to cardiovascular disease (CVD) [11–18]. Selective antioxidants such as quercetin, pomegranate juice, vitamin C and folic acid, vitamins C and E, resveratrol, and betaine have been shown to protect against CVD by restoring PON activity [11, 19–24].

Curcumin (diferuloylmethane), a derivative of the spice turmeric (*Curcuma longa*), has been used in traditional medicine for thousands of years [25]. It is known for its use in wound healing [26] and also as an antibacterial, antiviral, and antifungal agent [27]. Curcumin also has antioxidant and anti-inflammatory properties and prevents alcohol- or carbon tetrachloride-induced liver injury by inhibiting the activation of NF*κ*B signaling cascade, and the subsequent induction of proinflammatory cytokines, as well as chemokines, cyclooxygenase-2 (COX-2), and inducible nitric oxide synthase (iNOS) in Kupffer cells [28, 29]. A number of studies have shown that curcumin can reduce lipid peroxidation and liver and serum cholesterol in several liver injury models [30–32]. 

Therefore, in this present study, we have addressed for the first time the concerted protective role played by dietary curcumin on chronic ethanol and PUFA mediated adverse effects on hepatic liver injury due to oxidative stress as evidenced by alterations in liver (i) ROS, (ii) endogenous antioxidant, and reduced glutathione (GSH), as well as (iii) hepatic lipid score. In addition, we also evaluated the possible protective action of curcumin on the antiatherogenic gene, PON1/HTLase, as well as on plasma and liver lipids.

## 2. Materials and Methods

### 2.1. Animals

Female Wistar-Furth rats weighing between 130 and 150 g were purchased from Charles River Laboratories, Inc. (Wilmington, MA). Female animals were chosen because they are known to show more severe alcohol-induced liver injury than males [33–35]. They were housed in pairs per cage in plastic cages (40 × 24 × 18 cm), in a temperature-controlled room at 25°C with 12-hour light : dark cycle. All rats were fed a pelleted commercial diet (Purina Rodent Chow, #500, TMI Nutrition, St. Louis, MO) during the first week of acclimation period after arrival. They were then divided randomly into the following low *ω*-3 PUFA (LFO) and high *ω*-3 PUFA (HFO) groups as depicted in Table 1. Each of the LFO (*n* = 12) and HFO (*n* = 12) groups were further subdivided separately into three groups of 4 each: LFO or HFO (controls), LFO or HFO plus ethanol (LFOE or HFOE, 35% of dietary calories derived from ethanol), and LFOE or HFOE supplemented with curcumin 150 mg/day/kg [36] body weight (LFOEC or HFOEC). All LFO and HFO diets were made isoenergetic by substituting ethanol calories with dextrin-maltose and all these groups were pair-fed for 8 weeks. The composition of each diet (Dyets Inc., Bethlehem, PA) according to individual ingredients is described in Table 2.

### 2.2. Lipid Analysis

Blood samples were collected and centrifuged at 3100 rpm (Beckman J6M) for 10 min at 4°C. Separated serum, plasma, and liver samples were frozen at −80°C until assayed. Liver lipids were extracted as previously described [37]. Both plasma and liver lipids were determined using enzymatic reagents as described by the manufacturer (Teco Diagnostics, Anaheim, CA).

### 2.3. Quantification of Hepatosteatosis by Oil Red O

Livers from various experimental groups were cut into small pieces and washed immediately with ice cold PBS and mounted on optimum cutting temperature (OCT) embedding compound in peel-a-way embedding molds (Electron Microscope Sciences, Hatfield, PA). Liver tissues were cryosectioned and stained with oil red O to measure accumulation of lipid using an automated histometric system (Image-Pro Plus 6.1, Media Cybernetics, Bethesda, MD) as described previously [11]. The data are expressed as average oil red O percentage area of lipid staining. Values are means ± SEM.

### 2.4. 4-Hydroxynonenal (4-HNE) Staining of Liver Sections

Liver tissue sections were dewaxed in xylene and rehydrated and antigen was retrieved in heat incubated citrate buffer for 3 minutes. After washing in Tris buffered saline (TBS/T) containing Tween 20 (0.1%), sections were incubated overnight at 4°C in primary antibody raised in goat against 4-HNE (Millipore Incorporation, MA, USA) that was diluted to 1 : 100 in CAS-Block (Life Technologies Grand Island, NY) reagent. After an hour of secondary antibody incubation, a DAB Plus Substrate kit (Life Technologies) was used as the chromogen, and then slides were counterstained with hematoxylin. Intensity of 4-HNE-positive brown colored area was detected under optical microscopy (Observer Z1, Carl Zeiss Microimaging, Inc., Thornwood, NY) and % of brown pixels were calculated using AxioVision Rel.4.8.2 software in 4 randomly selected microscopic fields (magnification 40x) per section of all groups (*n* = 4).

### 2.5. Serum Biomarkers of Liver Injury

Leakage of liver transaminases into the blood compartment was assessed by measuring serum alanine aminotransferase (ALT) and aspartate aminotransferase (AST) in each animal from the various experimental groups using commercial kits according to manufacturer's instructions (Teco Diagnostics, Anaheim, CA). Mean serum ALT and AST activity in each group are expressed as international units per liter (IU/L) ± SEM.

### 2.6. RNA Isolation and Real-Time PCR

The total RNA was isolated from each liver using the Tri-Reagent (Molecular Research Center, Cincinnati, OH) as per manufacturer's instructions. Isolated total RNA was reverse transcribed by* in vitro* transcription as described by the manufacturer (Invitrogen, Carlsbad, CA). Quantitative real-time PCR was performed using a Bio-Rad iCycler using the SYBR green PCR mix (Bio-Rad, Hercules, CA). To amplify the coding region of PON1 and *β*-actin, we used gene-specific primers for rat PON1 forward primer 5′-TGCTGGCTCACAAGATTCAC-3′ and reverse primer 5′-TTCCTTTGTACACAGCAGCG-3′ and *β*-actin (forward: 5′-GTCAGGTCACTATCGGC-3′; reverse: 5′-CATGGATGCCACAGGATTCC-3′). Data were normalized to *β*-actin and then quantified. 

### 2.7. Serum PON1 and HTLase Activity Measurement

Serum PON1 enzyme activity was determined with paraoxon (Sigma-Aldrich Inc., St. Louis, MO) as the substrate. PON1 activity was measured as described by us previously [11, 13]. One unit of international enzyme activity is equal to 1 nmol of paraoxon hydrolyzed per min per mL serum. PON1 activity in each experimental group was expressed as percent of the activity in the corresponding control group.

Serum HTLase activity was measured using 10 mM *γ*-thiobutyrolactone (Sigma Inc.) as the substrate [11, 38] and Ellman's procedure to monitor the accumulation of free sulfhydryl groups via coupling with 1 mM 5,5′-dithiobis(2-nitrobenzoic acid). The assay conditions were the same as for PON1 assay above and the rate of *γ*-thiobutyrolactone hydrolysis was expressed as nmoles per mL per min.

### 2.8. Liver Reduced Glutathione (GSH) Measurement

1 g of tissue was weighed and washed with ice cold saline and homogenized in 10 mL of ice cold homogenizing buffer (10 mM Tris-HCl, pH 7.2; 250 mM Sucrose; 1 mM EDTA) containing 2x protease inhibitor cocktail. Reduced GSH was quantified according to manufacturer's instructions (Sigma Inc.).

### 2.9. Statistical Analysis

All data are presented as means ± standard error of the mean (SEM). The data were analyzed as one-way ANOVA. This design was selected rather than the 3-way factorial design in order to save unnecessary animals in the curcumin treatment control groups. Pairwise comparisons and linear contrasts were not made where the overall ANOVA *p* value exceeded 0.05. In order to protect against inflation of the type 1 error rate, a Bonferroni adjustment (SAS Institute Inc., Cary, NC, USA) was made to the critical alpha value that was selected by dividing the 0.05 alpha by the number of preplanned comparisons. A *p* value that was ≤ 0.05 was considered significant.

## 3. Results

### 3.1. Influence of Chronic Ethanol, Low *ω*-3 PUFA, High *ω*-3 PUFA, and Curcumin on Growth Pattern and Hepatosomatic Index in Various Experimental Groups

As shown in Table 3, animals from all experimental groups grew normally with significant gain in body weights, although the gains in the mean body weight in the ethanol fed groups were not as significant as in the corresponding control groups even when supplemented with curcumin. The mean liver weight relative to the mean body weight (hepatosomatic index) increased significantly by 23% (*p* < 0.0001), in the high HFOE group but not in the LFOE group.

### 3.2. Influence of Chronic Ethanol, Low *ω*-3 PUFA, High *ω*-3 PUFA, and Curcumin on the Status of Plasma and Liver Lipids

Table 4 shows that plasma triglycerides, VLDL-C, LDL-C, HDL-C, and total cholesterol were insignificantly altered in rats fed low PUFA alcohol group compared to control low PUFA group. LFOEC group showed significantly lowered plasma triglycerides compared to the LFOE group (*p* < 0.002); however plasma triglycerides significantly increased in HFOEC group compared to HFOE group (*p* < 0.04). HFOE group had significantly lower VLDL-C compared to HFO group (*p* < 0.0001). There was a tendency for plasma LDL-C, HDL-C, and total cholesterol to increase in HFOE group compared to HFOEC group, although the results were not statistically significant. Compared to respective control groups, both LFOE (*p* < 0.0001) and HFOE (*p* < 0.0005) groups exhibited significant increase in both liver triglycerides and cholesterol. Both liver triglycerides and cholesterol were significantly lower in LFOEC and HFOEC groups compared to the corresponding LFOE (*p* < 0.0001, *p* < 0.0001) and HFOE (*p* < 0.0001, *p* < 0.0001) groups, respectively.

### 3.3. Influence of Chronic Ethanol, Low *ω*-3 PUFA, High *ω*-3 PUFA, and Curcumin on Hepatosteatosis

**Figure 1:**
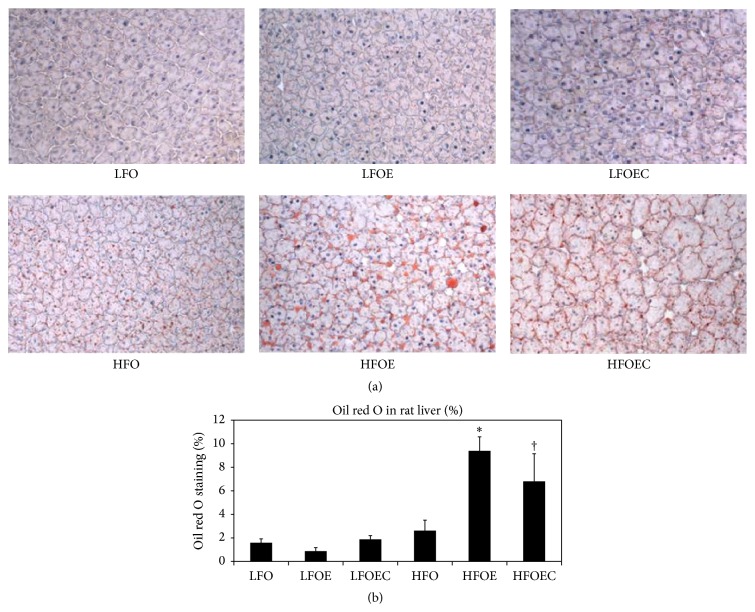
Histopathology of rat liver specimens and lipid deposits in the study groups. Cryosections of tissues were prepared as described in Section 2. Sections were stained with oil red O and counterstained with hematoxylin. (a) Representative 20x photomicrograph from the various groups. (b) Quantification of oil droplets in oil red O stained sections as determined by the Image-Pro Plus version 6.1 method. The data are expressed as average oil red O percentage area of lipid staining. Values are means ± SEM. Statistical significance of variance was calculated using* t*-test with Bonferroni correction; ^*∗*^ *p* < 0.0001 compared to all groups except HFOEC group; ^†^ *p* < 0.002 compared to HFOE group.

As shown in Figures 1(a) and 1(b), histochemistry of oil red O stained liver sections of low *ω*-3 PUFA ethanol fed rats showed no significant changes of fat deposition compared to control. Ethanol significantly increased hepatosteatosis in rats fed high *ω*-3 PUFA compared to other groups (*p* < 0.0001). Curcumin significantly reduced hepatosteatosis in HFOEC group compared to HFOE group (*p* < 0.002).

### 3.4. Influence of Chronic Ethanol, Low *ω*-3 PUFA, High *ω*-3 PUFA, and Curcumin on Liver Injury

**Figure 2:**
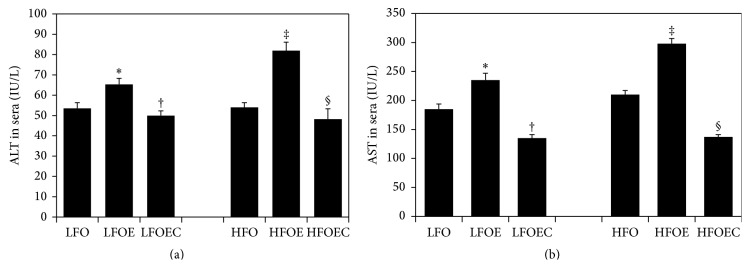
Influence of chronic ethanol, *ω*-3 PUFA, and curcumin on serum ALT and AST in rats fed low and high *ω*-3 PUFA diets. The animals in the indicated groups (*n* = 4) were pair-fed their respective Lieber-DeCarli alcohol containing liquid diets supplemented with the indicated concentration of curcumin for 8 weeks after which the animals were killed and each serum sample was analyzed. (a) ALT and (b) AST levels of these liver markers were measured as described in Section 2. The relative ALT and AST activities in the various experimental groups are expressed as international units per liter. The data are Means ± SEM. Statistical significance of variance was calculated using* t*-test with Bonferroni correction; ^*∗*^ *p* < 0.008 compared to LFO group; ^†^ *p* < 0.0002 compared to the corresponding LFOE group; ^‡^ *p* < 0.0001 compared to HFO group; ^§^ *p* < 0.0001 compared to HFOE group.

As shown in Figures 2(a) and 2(b), serum alanine aminotransferase (ALT) and aspartate aminotransferase (AST) did not alter significantly between LFO and HFO groups. However, while serum AST and ALT increased significantly by 28% (*p* < 0.008) and 40% (*p* < 0.008), respectively, in LFOE group compared to LFO group, these markers were significantly restored to the level of LFO group by curcumin supplementation (*p* < 0.0002). Furthermore, serum ALT and AST increased dramatically by 60% (*p* < 0.0001) and by 50% (*p* < 0.0001), respectively, in HFOE compared to the HFO group. Curcumin feeding significantly blocked these dramatic increases in serum ALT and AST as compared to HFOE group (*p* < 0.0001).

### 3.5. Influence of Chronic Ethanol, Low *ω*-3 PUFA, High *ω*-3 PUFA, and Curcumin on Liver PON1 mRNA Expression

**Figure 3:**
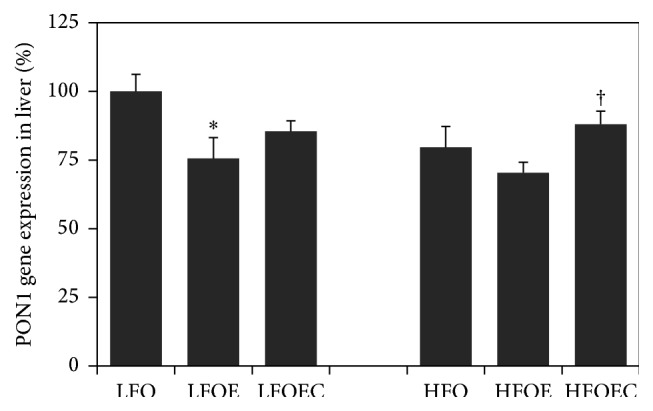
Effect of chronic ethanol and curcumin onPON1 mRNA expression in livers of rats fed low and high *ω*-3 PUFA diets. The animals in the indicated groups (*n* = 4) were pair-fed their respective Lieber-DeCarli control or alcohol containing liquid diets for 8 weeks after which the animals were killed and each liver was analyzed for PON1 mRNA as described in Section 2. The relative expression of PON1 mRNA in the HFO group is expressed as percent of the corresponding values in the LFO group. The data are Means ± SEM. Statistical significance of variance was calculated using* t*-test with Bonferroni correction; ^*∗*^ *p* < 0.0001 compared to LFO group; ^†^ *p* < 0.04 compared to HFOE group.

As shown in Figure 3, liver PON1 mRNA expression was decreased by 23% (*p* < 0.01) in the high *ω*-3 PUFA-fed group compared to the low *ω*-3 PUFA group. Chronic ethanol feeding decreased liver PON1 mRNA expression by 25% (*p* < 0.001) and 30% (*p* < 0.01) in both low and high *ω*-3 PUFA groups, respectively, compared to low *ω*-3 PUFA group. Curcumin significantly increased liver PON1 mRNA expression in high PUFA alcohol group (*p* < 0.04) but insignificantly increased PON1 mRNA in low PUFA alcohol groups.

### 3.6. Influence of Chronic Alcohol, Low *ω*-3 PUFA, High *ω*-3 PUFA, and Curcumin on Serum PON1 and HTLase Activity

**Figure 4:**
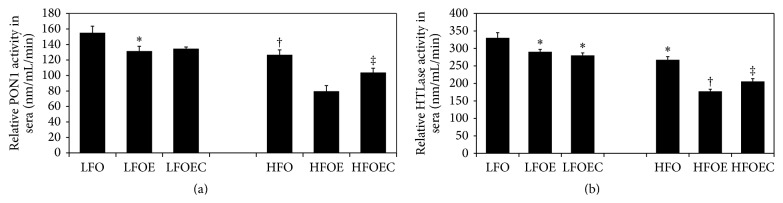
Influence of chronic ethanol and curcumin on serum PON1 and homocysteine thiolactonase activities were measured in rats fed low and high *ω*-3 PUFA diets. The animals in the indicated groups (*n* = 4) were pair-fed their respective Lieber-DeCarli alcohol containing liquid diets supplemented with the indicated concentration of the curcumin for 8 weeks after which the animals were killed and serum sample was analyzed. (a) Serum PON1 activity measured using paraoxon as substrate. (b) Serum homocysteine thiolactonase activity was measured using *γ*-thiobutyrolactone as substrate. Enzyme activities in the various experimental groups are expressed as percent of the corresponding values in the LFO group. The data are Means ± SEM. Statistical significance of variance was calculated using* t*-test with Bonferroni correction; ^**∗**^ *p* < 0.0001 compared to LFO group; ^†^ *p* < 0.0001 compared to LFO group; ^‡^ *p* < 0.05 compared HFOE group.

As shown in Figure 4, high *ω*-3 PUFA significantly decreased serum PON1 activity (Figure 4(a)) by 20% (*p* < 0.003) and HTLase activity (Figure 4(b)) by 28% (*p* < 0.0001, Figure 4(b)) compared to low *ω*-3 PUFA. Correspondingly, serum PON1 activity (Figure 4(a)) decreased by 23% (*p* < 0.05) in LFOE group and 58% (*p* < 0.0001) in HFOE group while serum HTLase activity (Figure 4(b)) decreased by 25% (*p* < 0.0001) and 59% (*p* < 0.0001) in the low and high *ω*-3 PUFA alcohol groups, respectively. Curcumin moderately stimulated serum PON1 activity by 15% (*p* < 0.05) (Figure 4(a)) in the high *ω*-3 PUFA alcohol group compared to HFOE group while it had no effect as compared to the LFOE group. Curcumin caused similar changes in serum HTLase activity only in the high *ω*-3 PUFA ethanol group.

### 3.7. Influence of Chronic Ethanol, PUFA, and Curcumin on Liver GSH

**Figure 5:**
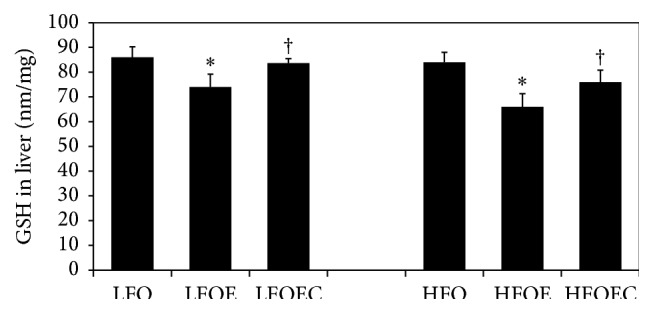
Influence of chronic ethanol, *ω*-3 PUFA, and curcumin on liver GSH level in rats fed low and high *ω*-3 PUFA diets. The animals in the indicated groups (*n* = 4) were pair-fed their respective Lieber-DeCarli alcohol containing liquid diets supplemented with the indicated concentration of curcumin for 8 weeks after which the animals were killed and each liver was analyzed for reduced GSH level in liver quantified as described in Section 2. The data are Means ± SEM. Statistical significance of variance was calculated using* t*-test with Bonferroni correction; ^*∗*^ *p* < 0.0001 compared to corresponding LFO or HFO group; ^†^ *p* < 0.005 compared to the corresponding LFOE or HFOE group.

As shown in Figure 5, there was no significant difference in the liver GSH levels in low or high *ω*-3 PUFA fed groups. However, chronic ethanol significantly decreased liver GSH levels by 27% (*p* < 0.0001) in low *ω*-3 PUFA group and by 38% (*p* < 0.005) in high *ω*-3 PUFA group. Curcumin restored liver GSH levels in both low and high *ω*-3 PUFA alcohol groups nearly to the control level (*p* < 0.05).

### 3.8. Influence of Chronic Ethanol, PUFA, and Curcumin on Lipid Peroxidation

**Figure 6:**
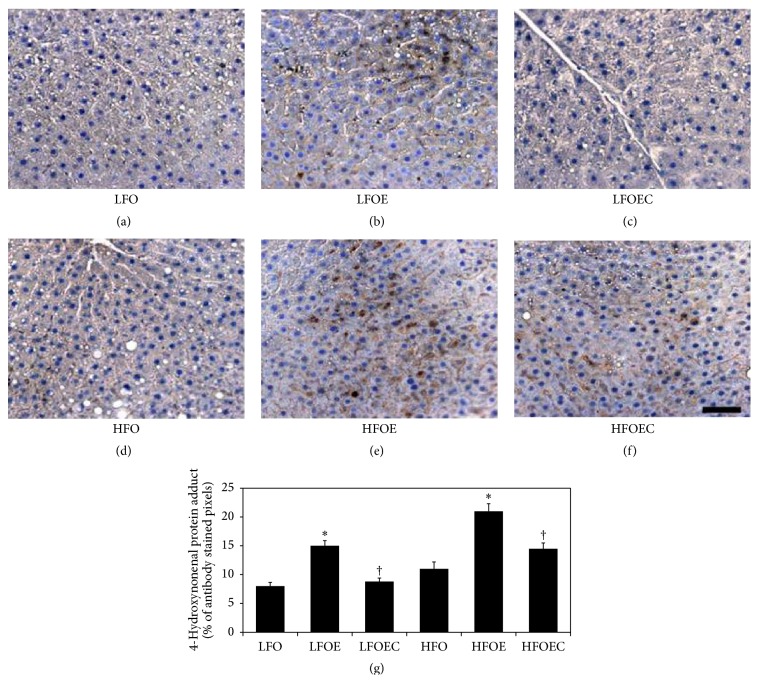
Effect of chronic alcohol, *ω*-3 PUFA, and curcumin on 4-HNE protein adducts levels (×200) in liver samples of various groups. The animals in the indicated groups (*n* = 4) were pair-fed their respective Lieber-DeCarli alcohol containing liquid diets supplemented with the indicated concentration of curcumin for 8 weeks after which the animals were killed and each rat liver sample was paraffin sectioned and stained with 4-HNE specific antibody. (1) Representative photomicrographs of immunohistochemically stained 4-HNE protein adducts (scale bars: 200 *μ*m) in liver sections of following groups shown in (a) LFO; (b) LFOE; (c) LFOEC; (d) HFO; (e) HFOE; and (f) HFOEC and (g). Bar graph shows the % of pixel values of brown color representing 4-HNE specific antibody reactivity from four randomly selected regions per section of each group (*n* = 4) measured using AxioVision Rel.4.8.2 software. The data are Means ± SEM. Statistical significance of variance was calculated using* t*-test with Bonferroni correction; ^*∗*^ *p* < 0.0001 compared to all groups except HFOEC group; ^†^ *p* < 0.001 compared to HFOE group.

To determine if the treatment with curcumin may have protected livers from the onset of alcohol-induced hepatitis, we determined levels of 4-HNE protein adduct which is an indicator of lipid peroxidation.  Figure 6 shows the staining of 4-HNE protein adducts was found to be significantly elevated in livers of LFOE (Figure 6(b)) and HFO (Figure 6(d)) and HFOE (Figure 6(e)) as compared to LFO (Figure 6(a)) group (*p* < 0.0001). Curcumin feeding significantly lowered levels of 4-HNE protein adducts in both LFOEC (Figure 6(c)) and HFOEC (Figure 6(f)) as compared to LFOE and HFOE groups (*p* < 0.001), respectively.

## 4. Discussion

This study clearly demonstrates the ability of dietary curcumin to significantly attenuate chronic ethanol and *ω*-3 PUFA mediated hepatomegaly (Table 3), hyperlipidemia (Table 4), hepatosteatosis (Figure 1), steatohepatitis (Figure 2), and antiatherogenic markers. Furthermore, the protective properties of curcumin were associated with its antioxidant actions. This was assessed by biochemical and histological methods.

Consistent with numerous alcohol feeding studies, the present study confirms that alcohol-fed animals do not seem to gain as much weight as the control animals in spite of pair-feeding leading to increased hepatosomatic index (Table 3). Strikingly, curcumin feeding seems to further reduce the gain in body weight resulting in greater increase in the hepatosomatic index in both a low and a high PUFA alcohol diet, showing its ameliorative effects on hepatomegaly. These results may be related in part to the ability of curcumin to suppress adipogenesis.

The significant decreases in plasma triglycerides in LFOEC group compared to LFOE group (Table 4) could be explained due to the potential inhibitory action of curcumin on hepatic triglyceride and VLDL synthesis. This is reflected in significant decreases in plasma VLDL-C and total cholesterol. The present work clearly shows that feeding a high PUFA alcohol diet increased hepatosteatosis (measured with oil red O staining) and liver triglycerides and total cholesterol (Table 4), whereas curcumin feeding significantly prevented these effects on the hepatosteatotic action of chronic ethanol. These results are in accord with the effects of curcumin in ob/ob male mice fed high fat diet [39].

Levels of ALT and AST were increased in animals fed with high PUFA alcohol (Figure 2), indicating hepatocellular injury and dysfunction. It must be pointed out that the gross liver appearance and liver histopathology as well as ALT and AST data show that the liver injury caused by chronic ethanol is markedly alleviated by curcumin feeding in high *ω*-3 fatty acid fed animals. Supporting our observations, curcumin has been shown to decrease hepatocellular injury and dysfunction in other hepatic injury models such as carbon tetrachloride [27, 36, 40], LPS/D-glucosamine [41], and methionine/choline deficiency [42].

Our present study clearly shows that feeding both low and high *ω*-3 PUFA significantly downregulates the gene expression and activity of the multifunctional antioxidant enzyme PON1 in both total liver and serum (Figure 3). These results are consistent with our previous study [11] and with those of Kudchodkar et al. [43]. Since chronic ethanol-induced cytochrome P4502E1 (Cyp2E1) exacerbates the accelerated generation of ROS in the presence of high *ω*-3 PUFA, it is reasonable that this increased ROS may be responsible for these deleterious effects on PON1 status that is vulnerable to oxidative stress. The ability of curcumin to partially restore chronic ethanol and *ω*-3 PUFA-mediated downregulation of serum PON1 activity and also PON1 mRNA in the liver as well as liver HTLase activity supports its antiatherogenic role of this antioxidant enzyme. Beneficial effects of other antioxidants such as pomegranate [44], vitamins C and E [22], and catechin [45] on serum PON1 activity are known. However, to our knowledge, this is the first time the protective effect of curcumin on chronic ethanol in combination with high *ω*-3 PUFA mediated decrease in serum PON1 activity has been demonstrated. Since serum PON1 is an integral component of serum HDL-C there is a direct positive correlation between serum HDL-C and serum PON1 level. Therefore, the inability of curcumin to completely restore serum PON1 and HTLase activities in HFOEC group to their corresponding levels in HFO group may be due to the fact that curcumin also independently lowers plasma HDL-C (Table 4). It is well known that, apart from plasma HDL, PON1 is the only other most important antiatherogenic marker because of its ability to not only prevent LDL oxidation, but also destroy the other atherogenic molecule, homocysteine [6–9, 13–20].

The above antihepatosteatotic and antiatherogenic effects of curcumin are accompanied by reciprocal restoration of severely depleted liver GSH level and a reduction of 4-HNE lipid peroxidation product in chronic ethanol-fed group. These results are consistent with previous reports [27, 40] indicating that curcumin acts as an antioxidant. Our data are also consistent with previous studies, which demonstrated that curcumin induced* de novo* synthesis of GSH [46, 47]. These results strongly suggest the antioxidant capacity of curcumin in nullifying ethanol-induced ROS generation.

## 5. Conclusion

In conclusion, we have demonstrated that dietary curcumin not only effectively protects against the deleterious effects of chronic ethanol in combination with high PUFA on liver injury, but also favorably improves the antiatherogenic markers such as PON1/HTLase and GSH. These findings raise the possibilities that consuming traditional natural antioxidants compounds like curcumin may benefit health by modulating the lipid metabolism in alleviating hepatosteatosis and suppressing atherogenesis.

## Figures and Tables

**Table 1:** Composition of various experimental diets, total fat, *ω*-3 PUFA, and ethanol.

Dietary groups (*n* = 4)	As percent of total calories			
	Total Fat	Ethanol	Protein	Carbohydrate
Low *ω*-3 PUFA control (LFO)	35^*∗*^	0	18	47
Low *ω*-3 PUFA + ethanol (LFOE)	35^*∗*^	36	18	11
Low *ω*-3 PUFA + ethanol + curcumin (LFOEC)	35^*∗*^	36	18	11

High *ω*-3 PUFA control (HFO)	35^†^	0	18	47
High *ω*-3 PUFA + ethanol (HFOE)	35^†^	36	18	11
High *ω*-3 PUFA + ethanol + curcumin (HFOEC)	35^†^	36	18	11

^*∗*^2.7% of total calories came from *ω*-3 PUFA; ^†^13.8% of total calories came from *ω*-3 PUFA.

**Table 2:** Composition of various diets according to individual ingredients.

Diet ingredient	Composition, grams/L			
	LFO	LFOE	HFO	HFOE
Casein	41.4	41.4	41.4	41.4
L-Cysteine	0.5	0.5	0.5	0.5
DL-Methionine	0.3	0.3	0.3	0.3
Menhaden oil	8.5	8.5	39.6	39.6
Olive oil	28.4	28.4	0	0
Safflower oil	2.7	2.7	0	0
Maltose dextrin	115.2	25.6	115.2	25.6
Cellulose	10	10	10	10
Mineral mix	8.75	8.75	8.75	8.75
Vitamin mix	2.5	2.5	2.5	2.5
Choline bitartrate	0.53	0.53	0.53	0.53
Xanthan gum	3	3	3	3

**Table 3:** Effect of ethanol and curcumin in low and high *ω*-3 PUFA on liver weight to body weight ratio.

Dietary groups (*n* = 4)	Initial body weight (g)	Final body weight (g)	Body weight gain (g)	Final liver weight (g)	Hepatosomatic index
LFO	130.1 ± 3.9	244.5 ± 5.9	114.4 ± 6.2	7.8 ± 0.4	3.2 ± 0.16
LFOE	160 ± 4.8	233.4 ± 4.8	73.4 ± 4.8^*∗*^	7.5 ± 0.3	3.2 ± 0.20
LFOEC	156.5 ± 3.4	209.0 ± 4.4	53 ± 4.1^†^	6.6 ± 0.7^†^	3.0 ± 0.24
HFO	159.4 ± 2.9	268.2 ± 7.3	108.8 ± 4.3^§^	8.4 ± 0.2	3.1 ± 0.18
HFOE	178.3 ± 4.2	228.3 ± 6.7	50.3 ± 4.6^*∗*^	8.8 ± 0.3^*∗*^	3.8 ± 0.21^*∗*^
HFOEC	157.4 ± 3.3	216.5 ± 0.6	59 ± 2.8^‡^	7.72 ± 0.1^†^	3.6 ± 0.26^*∗*^

Values are means ± SEM. Means in a column with different superscripts differ significantly (*p* < 0.05) as calculated by *t*-test with Bonferroni correction. ^*∗*^ *p* < 0.0001, compared to corresponding LFO or HFO groups; ^†^ *p* < 0.0001, compared to the corresponding LFOE or HFOE groups; ^‡^ *p* < 0.0001 compared to HFO; ^§^ *p* < 0.02 compared to LFO. Hepatosomatic index = (Liver weight × 100)/body weight.

**Table 4:** Effect of Chronic ethanol and curcumin on blood and livers lipids of rats fed low and high *ω*-3 PUFA diets.

Groups *n* = 4	Plasma (mg/dL)					Liver (mg/100 g)	
	Triglycerides	VLDL-C	LDL-C	HDL-C	Total-C	Triglycerides	Total-C
LFO	83 ± 5.5	17 ± 1.8	24 ± 2	26 ± 3	66 ± 8	132 ± 16	56 ± 6
LFOE	75 ± 4.5^*∗*^	15 ± 1.7	20 ± 4	25 ± 6	60 ± 6	146 ± 14	165 ± 8^*∗*^
LFOEC	64 ± 5^†^	13 ± 0.9	15 ± 3	20 ± 5	48 ± 7^†*∗*^	117 ± 12	117 ± 12^†*∗*^

HFO	104 ± 8^*∗*^	21 ± 1.3^*∗*^	6 ± 0.6^*∗*^	14 ± 2^†*∗*^	40 ± 7^*∗*^	549 ± 42^*∗*^	201 ± 8.0^†*∗*^
HFOE	74 ± 6.3^‡^	15 ± 1.5^‡^	11 ± 0.8^‡^	21 ± 4^‡^	47 ± 9	734 ± 49^‡^	393 ± 53^‡^
HFOEC	86 ± 7^‡,^	17 ± 2.0^‡^	10 ± 0.7^‡^	14 ± 2	40 ± 7	633 ± 55^†‡^	217 ± 5.0^†‡^

Values are means ± SEM. Means in a column with different superscripts differ significantly (*p* < 0.05) as calculated by *t*-test with Bonferroni correction. ^*∗*^ *p* < 0.05, compared to LFO; ^†^ *p* < 0.05, compared to the corresponding LFOE; ^‡^ *p* < 0.05 compared to the corresponding HFO.
